# Ginsenoside Rg3 Prevents Oxidative Stress-Induced Astrocytic Senescence and Ameliorates Senescence Paracrine Effects on Glioblastoma

**DOI:** 10.3390/molecules22091516

**Published:** 2017-09-10

**Authors:** Jingang Hou, Sunchang Kim, Changkeun Sung, Chulhee Choi

**Affiliations:** 1 Intelligent Synthetic Biology Center, Daejeon 34141, Korea; houjingang1225@126.com; 2 Department of Biological Sciences, Korea Advanced Institute of Science and Technology (KAIST), Daejeon 34141, Korea; 3 Department of Food Science and Technology, College of Agriculture and Biotechnology, Chungnam National University, Daejeon 34141, Korea; kchsung@kaist.ac.kr; 4 Department of Bio and Brain Engineering, Korea Advanced Institute of Science and Technology (KAIST), Daejeon 34141, Korea; cchoi@kaist.ac.kr

**Keywords:** astrocytic senescence, ginsenosides, SASP, brain tumor, inflammation

## Abstract

Senescent astrocytes in aging brain express senescence-associated secretory phenotype (SASP) and link with increased brain aging and its related diseases. In order to determine whether ginsenosides ameliorate the astrocytic senescence in vitro, human astrocytic CRT cells and primary rat astrocytes were used in the present study. Ginsenosides Rg1, Re, Rb1 and Rg3 (5 μg/mL) could effectively prevent the astrocytic senescence induced by H_2_O_2_ exposure. However, these ginsenosides did not reverse the astrocytic senescence. Importantly, senescent astrocytes herein produce SASP. The expression of major components of SASP, IL-6 and IL-8, are greatly increased in senescent astrocytes. Ginsenoside Rg3 (10 μg/mL) effectively suppressed the expressions of IL-6 and IL-8, which is associated with regulations of NF-κB and p38MAPK activation. In addition, after incubation with Rg3, conditioned medium from senescent astrocytic CRT cells significantly decreased the ability to promote the proliferation of astrocytoma U373-MG, U87-MG and U251-MG cells compared with non-treated senescent samples. Similar patterns were confirmed in chemotherapy-induced glioblastoma senescent cells. The present study explored a potential candidate for amelioration of astrocytic senescence and SASP in brain aging, which provided a basis for developing strategies to reduce the dark side of senescence in normal or pathological aging process.

## 1. Introduction

Aging is characterized by a progressive loss of physiological function, giving rise to tissue dysfunction and increased vulnerability to diseases and death. The hallmarks of aging are summarized to identify pharmaceutical targets and improve human healthy lifespan. One of the these hallmarks is cellular senescence, which have a complex phenotype characterized by irreversible cell-cycle arrest mediated predominated by p16^ink4a^ and p21, increased cellular size, altered morphology, resistance to apoptosis, and a unique secretory phenotype referred as senescent-associated secretory phenotype (SASP) [1]. Although researches on the causes and mechanisms underlying the various types of cellular senescence are still in its infancy, telomere erosion, DNA lesion and reactive oxidation species (ROS) have been shown to induce cellular senescence [2,3,4,5]. The damaging ROS known to be responsible for neurotoxicity are hydrogen peroxide (H_2_O_2_), superoxide anions (O_2_^−^) and hydroxyl radicals (OH). H_2_O_2_ is the major precursor of ROS that may result in cellular damage and its excessive accumulation is observed during brain aging and neurodegenerative diseases [6]. Hence H_2_O_2_ is an available stress inducer for researches on cellular senescence and the aging brain.

Astrocytes play a fundamental role on the maintenance of homeostasis of the central nervous system (CNS) as well as defense [7]. Aging brain affects many functional characteristics that are regulated by astrocytes. Increased expression of glial fibrillary acidic protein indicates human astrocyte senescence, which also implies this astrocytic senescence represses their enhancement on neurogenesis and neuronal protection [8,9]. In aging rat brain, expression of cytokines, such as TNF-α, IL-1β and IL-6 were localized in astrocytes, suggesting astrocytes express a proinflammatory phenotype during aging [10]. In culture systems, astrocytes initiate senescence progress in response to oxidative stress, proteasome inhibition and exhausted replication. Prominent increased senescence markers were observed and it also implies that astrocytes are more susceptible than fibroblast in response to oxidative stress [11]. Additionally, depletion of glutathione could trigger inflammatory pathway and increase secretion of IL-6, demonstrating a neurotoxicity of secretory astrocytic phenotype in relevant to aging and degenerative neurological disease [12]. Extended culture of astrocytes associates with increased expression of senescence markers and loses the neuroprotective capacity [13]. Interestingly, β-amyloid peptide also stimulates chemokine expression, the secretion of ROS and cytokines from rat astrocytes [14]. All of above observations indicate that at least in vitro astrocytes are capable of triggering senescence phenotype and the astrocytes in aged brain display the characteristic typical for inflammatory SASP.

In oriental medicine, ginseng is able to strengthen vital energy in different body locations to delay aging. Interestingly, ginseng are also viewed as adaptogenic herbs, giving that they increase body’s resistance to various stresses, trauma, anxiety and fatigue. Ginsenosides, the metabolites of ginseng, are attributable to the pharmacological and biological activities of ginseng used as medicinal plants in traditional oriental medicine [15]. Unsurprisingly, a large number of researches encompassed anti-aging of ginsenosides emerges every year. Especially, the beneficial effects of ginsenosides on aging related pathologies, such as Alzheimer Disease, Parkinson disease and Huntington’s Diseases are reported [16,17,18]. Hence, the non-specific and multi-targets properties of ginsenosides are proposed as typical characteristics of anti-aging herbal ginseng.

In the present study, we used oxidative stress stimulated by H_2_O_2_ to induce astrocytic senescence. Our results demonstrate that Rg3 is able to prevent astrocytic senescence, and further ameliorate the side effects of SASP on brain pathology.

## 2. Results

### 2.1. Selected Ginsenosides Prevent Astrocytic Senescence and Decrease SASP

**Figure 1:**
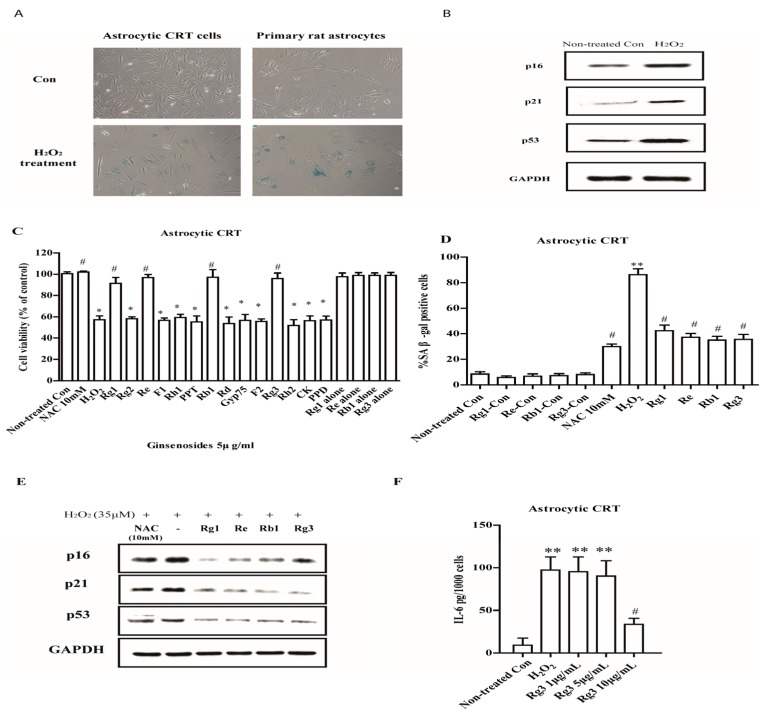
Ginsenosides prevent astrocytic senescence and decrease SASP induced by H_2_O_2_. (**A**) Senescent astrocytic cells and primary rat astrocytes induced by H_2_O_2_ express SA-β-gal under light microscopy. (**B**) Proteins were extracted from control and H_2_O_2_ treated astrocytic CRT cells and analyzed by western blotting for senescent markers p16, p21 and p53. GAPDH as a loading control. (**C**) Astrocytic CRT cells were pretreated with 5 μg/mL of ginsenosides for 12 h, followed by 2 h treatment with 35 μM of H_2_O_2_, and then cell proliferation was assayed after 72 h by WST-1. * *p* < 0.05 versus H_2_O_2_-treated group. (**D**) The percentage of SA-β-gal positive cells in astrocytic cells after treated by indicated groups. 10 μM of NAC was used as positive control. (**E**) Protein levels of senescence markers p16, p21 and p53 in astrocytic CRT cells of “D”. GAPDH as a loading control. (**F**) Major components SASP, IL-6 quantified using ELISA. * *p* < 0.05, ** *p* < 0.01, groups versus Non-treated Con; ^#^ *p* < 0.05, groups versus H_2_O_2_. Five samples corresponding to each indicated ginsenoside group were evaluated for cell viability and galactosidase staining. Three samples corresponding to each concentration were counted.

To establish the cellular senescence in response to oxidative stress in human astrocytes, 35 μM and 150 μM of H_2_O_2_ were used to treat CRT astrocytic cells and primary rat astrocytes respectively for 2 h, then the medium were replaced and cultured for 72 h. The results showed that H_2_O_2_ treatment induced the expressions of SA β-gal and resulted in morphologic changes in CRT cells and primary rat astrocytes. The cultures displayed enlarged, flatten morphology typical of senescence, and entered a growth arrest (Figure 1A). These observed changes were accompanied by increased expression of p16, p21 and p53, three biomarkers of senescence (Figure 1B). To test the possibility that ginsenosides could regulate astrocytic senescence, astrocytic cells were exposed to 14 different ginsenosides. Selected ginsenosides Rg1, Re, Rb1 and Rg3 with 5 μg/mL pretreatment for 12 h significantly decreased cell growth inhibition (Figure 1C) and SA β-gal expression (Figure 1D) in CRT astrocytic cells. These selected ginsenosides also suppressed the expressions of p16, p21 and p53 in H_2_O_2_ treated CRT cells (Figure 1E).

To determine whether selected ginsenosides decrease the expressions of IL-6, the major SASP component in human cells, we measured the extracellular expression of IL-6 using ELISA assay. Interestingly, newly selected ginsenoside Rg3 decreased the secretion of IL-6 when added after the SASP fully developed (8 d after H_2_O_2_ induced senescence; Figure 1F).

### 2.2. Ginsenoside Rg3 Decreases SASP by Suppressing NF-κB Nuclear Translocation and p-38 Activation

**Figure 2:**
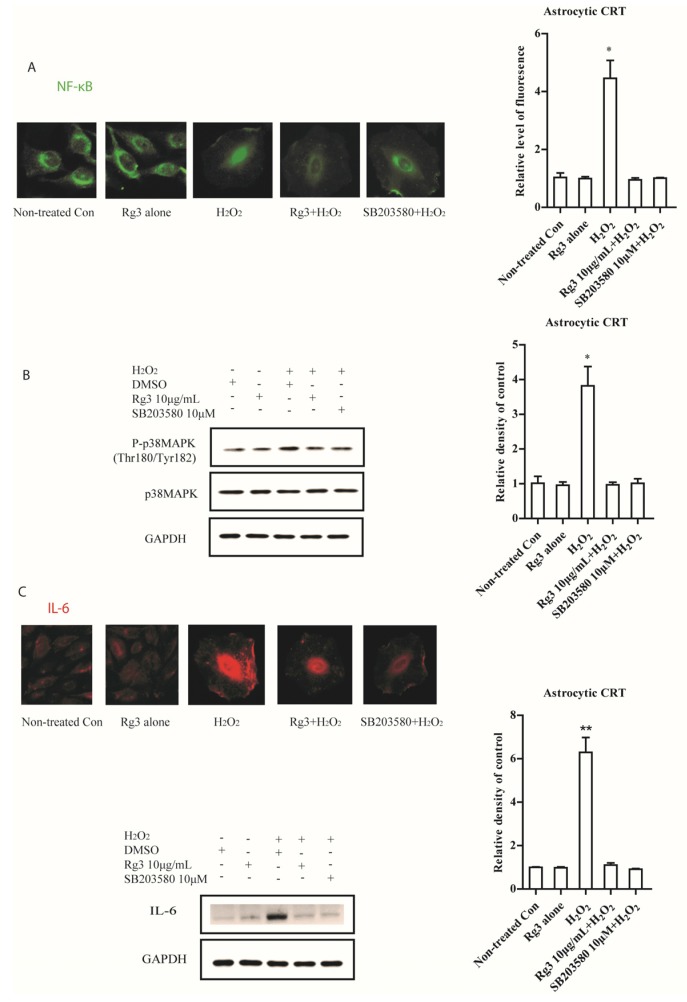
Senescent astrocytic CRT cells develop SASP by activating NF-κB nuclear translocation and p38MAPK activity. (**A**) Immunofluorescence with NF-κB in senescent astrocytic CRT cells. H_2_O_2_ (35 μM) treatment after 8 d induced flatten morphological changes and profound NF-κB nuclear translocation. Ginsenoside Rg3 (10 μg/mL) treatment significantly decreased this translocation. (**B**) Western blots showing the total and phosphorylated p38 MAPK in H_2_O_2_ (35 μM) treated senescent astrocytic CRT cells. 10 μM SB203520 as a positive control. GAPDH as a loading control. (**C**) The expressions of IL-6 were abolished in the presence of SB203580 or Rg3 in senescent astrocytic CRT cells. * *p* < 0.05, ** *p* < 0.01, groups versus Non-treated Con.

As NF-κB stimulates the transcription of many SASP genes [19], we tested the possibility that selected ginsenosides decreased NF-κB activity. In addition, p-38 mitogen-activated protein kinase (p38MAPK), a mediator of SASP in response to various stimuli [20], were also measured. Intriguingly, we measured the nuclear translocation of NF-κB by immunocytochemistry staining. Selected ginsenoside Rg3 reduced NF-κB nuclear translocation (Figure 2A). Similarly, activation of p38MAPK significantly increased during senescence process, nevertheless, selected ginsenoside Rg3 suppressed this activation in senescent astrocytic CRT cells (Figure 2B). Furthermore, we found that this suppression on p38MAPK greatly abolished the expression of IL-6 (Figure 2C). 

### 2.3. Selected Ginsenosides Do Not Reverse Cellular Senescence

**Figure 3:**
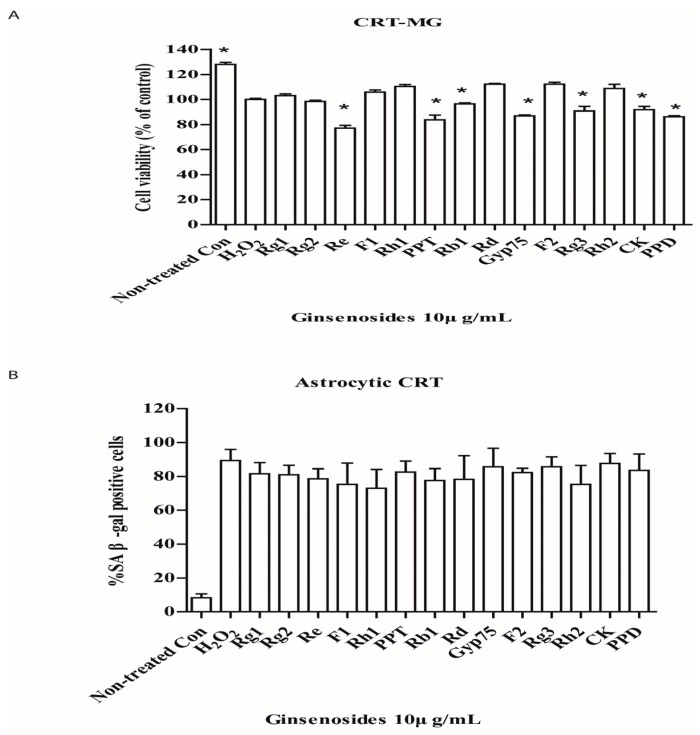
Ginsenosides do not reverse astrocytic senescence. (**A**) Senescent astrocytic CRT cells were treated with 10 μg/mL of ginsenosides for 24 h, then cell viability was assayed by WST-1. Cell death was observed after treatment. * *p* < 0.05 versus H_2_O_2_ group. (**B**) The percentage of cells expressing SA-β-gal was determined by light microscopy and counting. Five samples corresponding to each indicated ginsenoside group were evaluated for cell viability and galactosidase staining.

In the senescent human astrocytic CRT cells (4 d after senescence induction), post-treatment of ginsenosides did not reverse the cell growth arrest induced by H_2_O_2_ (Figure 3A), nor did it decrease the percentage of cells that robustly expressed senescence-associated β-galactosidase (Figure 3B). In addition, ginsenosides seemed to selectively induce cell death. We proposed that certain ginsenosides are potential senolytic agents for senescent astrocytes induced by H_2_O_2_. Although selected ginsenoside greatly decreased the expressions of IL-6 which are reported to be responsible for senescence growth arrest, it does not reverse this arrest. We presume that other factors derived from SASP are attributed to this phenomenon. Further tests are needed to prove it. 

### 2.4. Ginsenoside Rg3 Decreases the Ability of Senescent Astrocytes to Inhibit Normal Cell Growth

**Figure 4:**
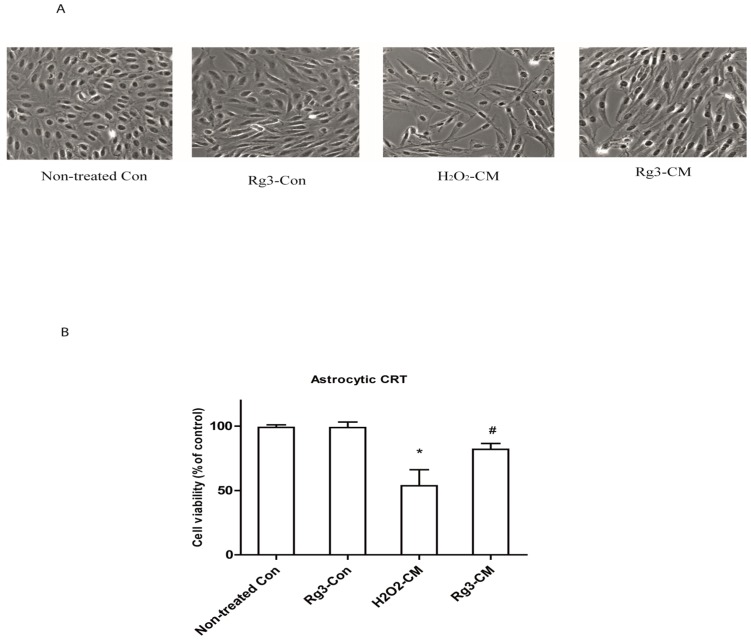
Ginsenoside Rg3 suppresses cell growth arrest by conditioned medium from senescent astrocytic CRT cells. (**A**) Conditioned medium (CM) inhibited the growth of astrocytic CRT cells after 48 h treatment. Cells treated with conditioned medium showed enlarged, flattened, and irregular shape. The morphological changes were observed under light microcopy. (**B**) Cell proliferations were determined by WST-1. * *p* < 0.05, Non-treated Con versus H_2_O_2_-CM; ^#^ *p* < 0.05, ginsenoside Rg3-CM versus H_2_O_2_-CM. Five samples corresponding to each indicated ginsenoside group were evaluated for cell viability.

The main SASP components, IL-6 can reinforce the cell growth arrest in oncogene-induced senescent cells [21]. As can be remarkably seen in Figure 4, conditioned medium reduced cell growth of normal astrocytic CRT cell by 50%. However, conditioned medium from ginsenoside Rg3 group significantly rescued the cell growth up to 27%. Interestingly, Rg3 treatment did not rescue the morphological changes induced by conditioned medium.

### 2.5. Oxidative Stress-Induced Astrocytic Senescence Secrets High Level of IL-8 

**Figure 5:**
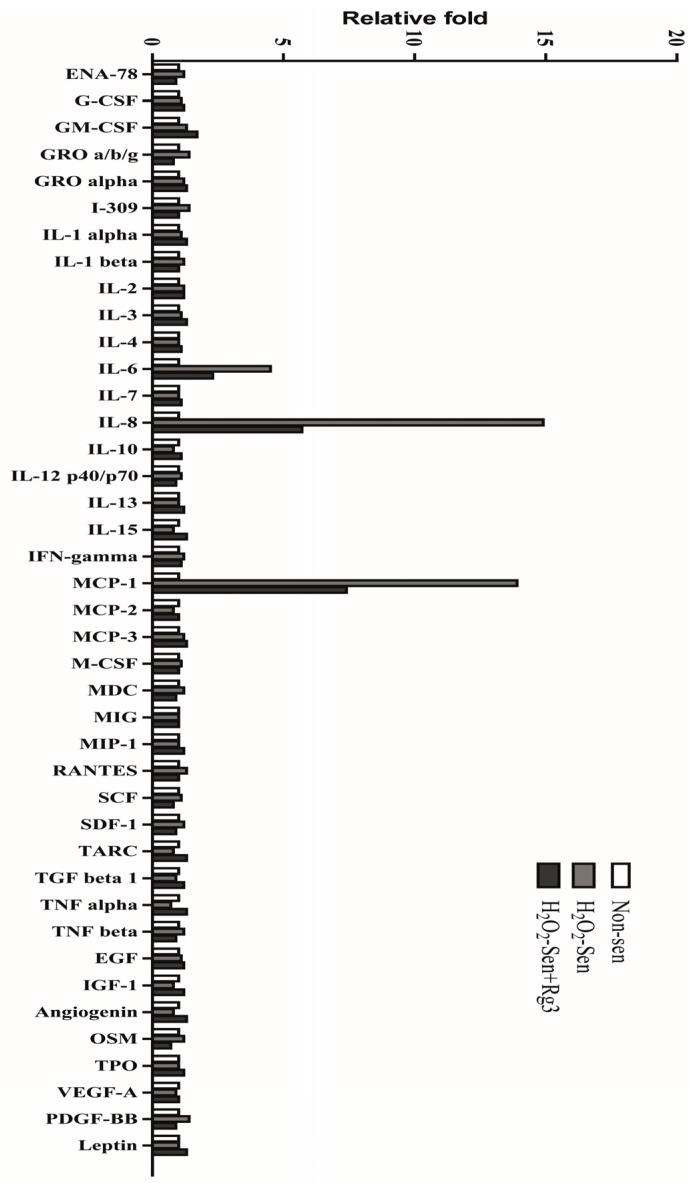
Ginsenoside Rg3 suppresses the secretion of major components from SASP. Conditioned medium from non-senescent or senescent cells treated with or without Rg3 were collected and analyzed by antibody array.

In order to determine whether senescent astrocytic cells secrete other SASP components, the conditioned medium by antibody array test was examined. Intriguingly, we found that IL-8 and MCP-1 were secreted substantially in senescent astrocytic CRT cells. Importantly, Rg3 could suppress the IL-8 and MCP-1 secretion, which is similar to the suppression of IL-6 secretion (Figure 5). 

**Figure 6:**
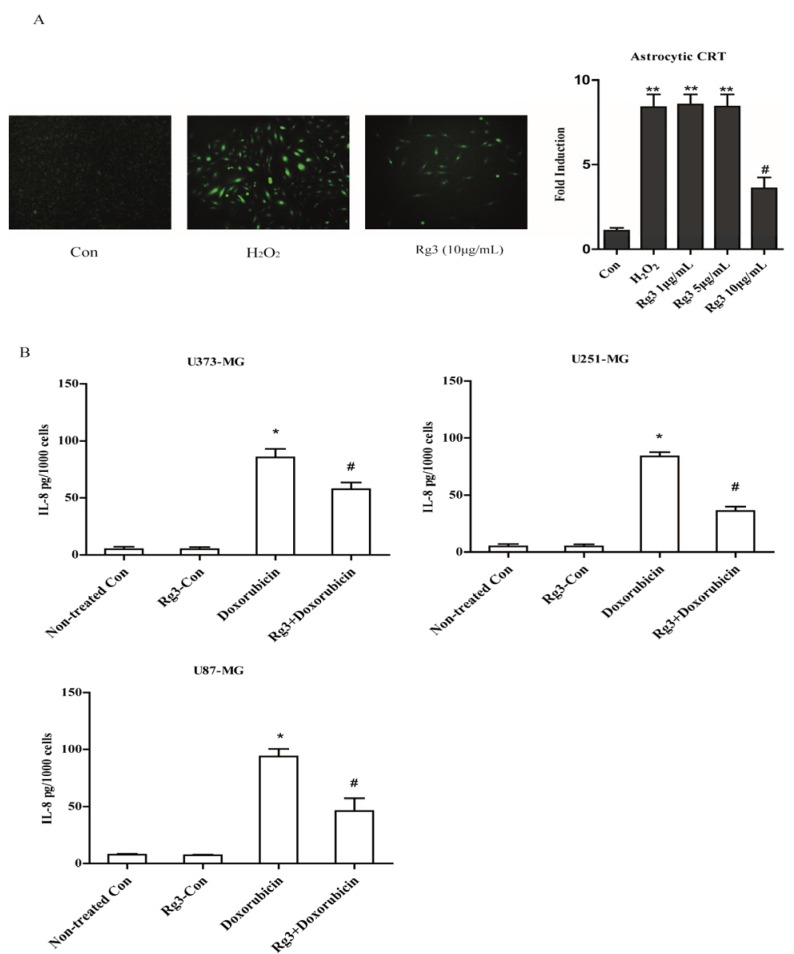
Oxidative stress induced astrocytic senescence and secreted high level of IL-8. (**A**) The transcriptional level of IL-8 was evaluated by reporter constructed CRT (IL-8p-d2EGFP) cells. (**B**) Doxorubicin (100 nM) treatment for 72 h induced astrocytic senescence and secretion of IL-8. * *p* < 0.05, Non-treated Con versus Doxorubicin-CM; ^#^ *p* < 0.05, ginsenoside Rg3-CM versus Doxorubicin-CM. Five samples corresponding to each indicated ginsenoside group were evaluated for cell viability and galactosidase staining. Three samples corresponding to each concentration were counted.

To test whether Rg3 regulates IL-8 expression at transcriptional level, astrocytic CRT cells were stably transfected with construct IL-8p-d2EGFP, which is a destabilized enhanced green fluorescent protein (EGFP)-expressing plasmid under the control of the IL-8 promoter. The cells were induced to senescence as indicated in method section. As a result, 7.1 fold increase by H_2_O_2_ was observed compared with control group. Additionally, Rg3 (10 μg/mL) reduced the IL-8 EGFP reporter activity by 2.6 fold compared with control group (Figure 6A). 

To confirm if Rg3 could selectively suppress the expression of IL-8 in oxidative stress-induced SASP, we measured the IL-8 secretion in senescent glioblastoma cells induced by chemotherapy agent Doxorubicin. Notably, Rg3 significantly decreased IL-8 secretions in U373-MG, U251-MG and U87-MG cell lines (Figure 6B). 

### 2.6. Ginsenoside Rg3 Decreases Paracrine Effects of Senescent Astrocytic Cells on Glioblastomas

**Figure 7:**
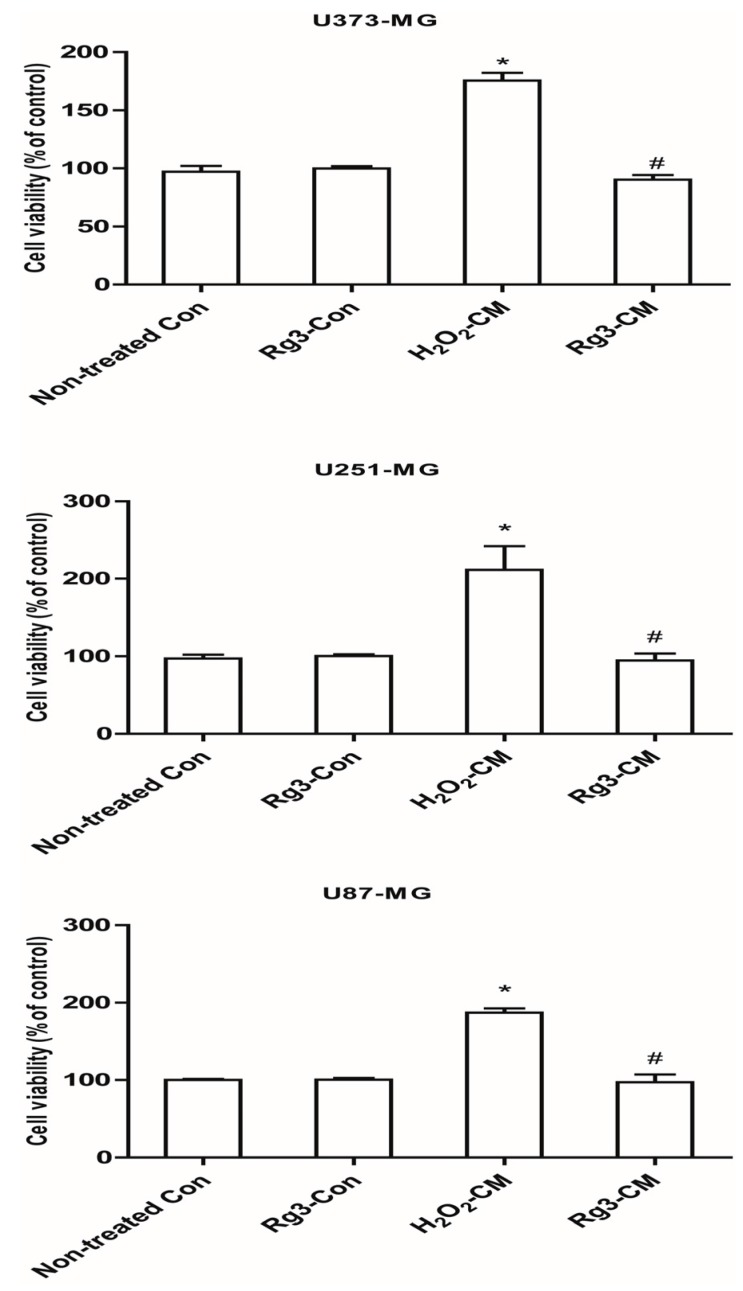
Conditioned medium promotes the cellular proliferation of major glioblastoma cell lines. The indicated glioblastoma cells U87-MG, U373-MG and U251-MG were incubated with conditioned medium for 48 h. Cell proliferations were determined by WST-1. * *p* < 0.05, group versus Non-treated control; ^#^ *p* < 0.05, ginsenoside Rg3-CM versus H_2_O_2_-CM. Five samples corresponding to each indicated ginsenoside group were evaluated for cell viability.

SASPs were able to stimulate cell proliferation and tumorigenesis, hence we determined the effects of SASP from senescent astrocytes on glioblastomas. Unsurprisingly, all the three glioblastoma cell lines (U373-MG, U87-MG and U251-MG) showed essential increased proliferation. Rg3 greatly reduced the stimulation of proliferation effect of conditioned medium from senescent astrocytic CRT cells (Figure 7). Additionally, Rg3 alone treatment did not differ from non-treated group.

## 3. Discussion

Our results confirmed that oxidative stress induces astrocytic senescence and possible brain tumorigenesis process that involves SASP. Emerging evidences indicate that cellular senescence accumulates in tissues with aging, which confers tissue degeneration and dysfunction [22,23]. The SASP that implies chronic inflammation in brain may be partly explained by senescent astrocytes. Our findings show that some selected ginsenosides (Rg1, Re, Rb1 and Rg3) can prevent the astrocytic senescence induction by oxidative stress, however, these ginsenosides do not reverse senescence. It is possible, therefore, that manipulations on SASP by potential ginsenosides may provide a new strategy to abrogate the deleterious effects of astrocytic senescent in brain. Intriguingly, one anticipated finding was that ginsenoside Rg3 inhibits expressions of IL-6 and IL-8, major components of SASP, by inactivating NF-κB and p38MAPK activity. This finding was unexpected because NF-κB and p38MAPK activations have been extensively investigated and reviewed in senescence, and suggests the potential mechanism of Rg3 on regulating astrocytic SASP.

Additionally, glioblastoma, a kind of aggressive and fatal brain tumor increases with age [24]. IL-6 secretion in response to external stimuli or intrinsic factors, such as IL-1β or TNF-α, was reported previously [25]. Furthermore, IL-6-induced activation of STAT3 have been wildly reported to promote invasion and migration in U251-MG and U87-MG glioblastoma cells [26]. Importantly, IL-6 derived from cerebral cells, strongly inhibits apoptosis of glioblastoma cell invasion and plays a pivotal role in maintain glioblastoma stem cell properties [27]. Additionally, IL-8 is highly expressed in glioblastoma cell lines and linked to malignant cell proliferation and tumor growth [28]. More importantly, patients received surgical removal of GBM commonly take a long-term radiation and chemotherapy treatment. It is reported that long-term radiation induces upregulation of IL-6 and IL-8 in tumor cells [29] and treatment with temozolomide increases production of inflammatory mediators in brain [30]. In the present study, our results further confirmed inhibition of IL-6 and IL-8 secretion of conditioned media from senescent astrocytic CRT cells could abrogate the stimulation of astrocytoma cell lines (U373-MG, U87-MG and U251-MG). This abolishing action was also available in chemotherapy induced glioblastoma cellular senescence. Hence these findings are significant in at least two major respects: (1) SASPs form senescent astrocytes secrete IL-6 and IL-8 (2) IL-6 and IL-8 promote proliferation of glioblastoma. This combination of findings provides some support for the conceptual premise that cellular senescence may be potential target for curing aging-related pathologies in brain. 

Ginsenoside Rg3 is widely known as a clinical anticancer therapy and enriched in ginseng products, such as red ginseng and black ginseng [31]. Recent studies also suggest that ginsenoside Rg3 greatly sensitizes cancer cells when combined with conventional chemotherapeutic agents [32,33]. Moreover, chemotherapy is reported to induce senescence and this promotes side effect in tissue and even cancer relapse [34]. Our results herein suggest another possibility, at least in vitro human astrocyte models: Ginsenoside Rg3 may decrease inflammation caused by senescent astrocytic cells and the SASP. In addition, it is worthy that ginsenoside Rg3 has been implicated to induce cancer cell death [35], as well as protection of normal cells (astrocytes) from senescence. 

Although ginsenoside Rg3 has been extensively reported to exert anticancer pharmacological activities [36,37], it barely targeted brain tumors both in vivo and in vitro [38]. The ultimate limitation of Rg3 on brain tumor might be attributed to the poor blood brain barrier permeability. Thus, efficient delivery methods are needed with regard to target brain tumor and senescent brain cells. Exosomes, secreted nanoparticles, are important mediators of intercellular communication and proposed as drug delivery vehicles [39]. Therapeutic cargos (such as, siRNA, peptide, doxorubicin and curcumin) are loaded into exosomes to target various diseases [40,41,42,43]. This possible source of delivery system could be a carrier for ginsenoside Rg3 to reach the area of disease. 

Here, our findings suggest that selected ginsenosides Rg1, Re, Rb1 and Rg3 partly prevent the senescence and Rg3 greatly suppresses the major components of SASP, IL-6 and IL-8. Therefore, ginsenoside Rg3 might be a candidate for the amelioration of senescence and its inflammatory or paracrine side effects during aging process. This may help us to understand how ginsenosides may suppress aging and its related pathologies, including late-life cancer process. Importantly, ginsenoside Rg3 may provide a candidate for declining the dark side of senescence derived from normal or chemotherapy-induced aging process. 

## 4. Materials and Methods 

### 4.1. Cells and Culture Conditions 

CRT-MG and U373-MG cells were maintained in RPMI 1640 medium (Hyclone, South Logan, UT, USA) with 10% heat-inactivated fetal bovine serum (FBS, G), 100 U of penicillin/mL, and 100 μg of streptomycin/mL (Thermo Fisher Scientific Korea Ltd., Seoul, Korea) as previously described [44]. U251-MG and U87-MG cells were grown in Dulbecco’s Modified Eagle Medium (DMEM, Hyclone, South Logan, UT, USA) supplemented with 10% heat-inactivated fetal bovine serum (FBS), 100 U of penicillin/mL, and 100 μg of streptomycin/mL. Primary rat and human astrocytes were maintained in 10% FBS-DMEM containing 1% nonessential amino acids (Thermo Fisher Scientific Korea Ltd., Seoul, Korea). Stable cell line CRT-MG/IL-8p-d2EGFP cells were prepared and maintained as previously described [45]. Briefly, stable reporter cell lines transfected with the human IL-8 promoter-luciferase reporter construct were generated. Then the construct and a destabilized enhanced green fluorescent protein (EGFP)-expressing plasmid, pd2EGFP (Clontech, Mountain View, CA, USA), were digested by using XhoI and BamHI (Clontech, Mountain View, CA, USA). The pd2EGFP backbone and the 546-bp insert containing the IL-8 promoter were ligated to generate IL-8p-d2EGFP. 

### 4.2. Reagents

Ginsenosides Rg1, Re, F1, Rh1, Rh1, PPT, Rb1, Rd, Gyp75, F2, Rg3, Rh2, CK and PPD, with a purity of more than 98%, were all prepared with High Performance Liquid Chromatography (HPLC) (Tokyo, Japan). Each ginsenoside was dissolved in dimethyl sulfoxide (DMSO) as 10 mg/mL solution. *N*-acetyl cysteine (NAC) and hydrogen peroxide (H_2_O_2_) were purchased from Sigma (St. Louis, MO, USA). 

### 4.3. Senescence Induction

Induction of astrocytes senescence by H_2_O_2_ was performed as previously described [11], CRT-MG cells and primary rat astrocytes were incubated for 2 h in medium containing 35 and 150 μM H_2_O_2_ (determined by preliminary tests) respectively, then washed and cultured by fresh complete medium for 3 d.

### 4.4. SA-β-Gal Staining

Senescence-associated β-galactosidase (SA-β-gal) staining was performed using a SA-β-gal kit (#9860, Cell Signaling Technology, Inc., Beverly, MA, USA) in accordance with the manufacturer’s protocol. The cells were fixed for 10–15 min at room temperature, then rinsed twice with PBS and stained with staining solution at a final pH of 6.0 for at least overnight. The SA-β-gal positive cells develop blue color and were counted under a phase-contrast microscope. The experiment was repeated three times in each group.

### 4.5. Preparation of Conditioned Medium (CM), Antibody Array Test and Quantification of IL-6 and IL-8 Secretion

CRT-MG cells were exposed by H_2_O_2_ (35 μM) to induce premature secretory senescence. Eight days following senescence induction, fresh medium containing ginsenoside Rg3 or SB203580 or DMSO were added and cultured for 24 h. Then replaced by serum-free and collected from both pre-senescent and senescent cells after 48 h. CM were centrifuged for 20 min at 5000 rpm, and filtered through 0.22 μm bottle-top filters (Sartorius Stedim Biotech, Göttingen, Germany). The collected medium were applied to antibody array (Ray Biotech, Cat#AAH-CYT-5-2, Norcross, GA, USA). IL-6 and IL-8 levels in conditioned media were quantified using Human IL-6 and IL-8 ELISA RayBiotech protocol (Cat# ELH-IL-6-1, Norcross, GA, USA)

### 4.6. Cell Viability Assay

Cell viability was evaluated by the WST-1 assay (Abcam, Cambridge, MA, USA), which is based on the enzymatic cleavage of the tetrazolium salt WST-1 to formazan by cellular mitochondrial dehydrogenase present in viable cells. In brief, after 24 h treatment, 20 μL of WST-1 was added to each well and the plates were incubated at 37 °C for 2 h. The plates were then centrifuged and 100 μL of the medium was withdrawn for measuring the absorbance value at a wavelength of 450 nm using microplate reader. 

### 4.7. Immunoblotting

To assay the expression of p16^ink4a^, p53, p21, total cellular proteins were extracted using M-PER mammalian protein extraction reagent (Thermo Fisher Scientific Korea Ltd., Seoul, Korea). Briefly, cells grown in 60 mm petri dishes were washed three times with 5 mL cold PBS. A volume of 200 μL of extraction reagent was added to each plate. The cells were then dislodged by scraping and were transferred to Eppendorf tubes. The protein concentrations were estimated and equalized. The cell lysate was loaded in each lane and separated by SDS-PAGE. Proteins were electrophoretically transferred to nitro-cellulose membrane and non-specific sites were blocked by 3% of Bovine Serum Albumin (BSA). Protein expression was detected using a primary antibody p16^ink4a^, p53, p21 (1:2000 Abcam, Cambridge, MA, USA), GAPDH (1:4000, Santa Cruz, Dallas, TX, USA) and horseradish peroxidase-conjugated anti-rabbit and anti-mouse secondary antibodies (1:5000, Santa Cruz, Dallas, TX, USA). Quantitative analysis of Western blot was performed using Image J software (Windows version of NIH Image, http://rsb.info.nih.gov/nih-image/, USA). The protein of GAPDH was used as the loading control. 

For immunofluorescence assay, the cells were first rinsed twice with PBS, followed by fixation with 4% (*wt*/*vol*) paraformaldehyde for 20 min. Cells were blocked in assay diluent containing 3% (*wt*/*vol*) BSA and 0.15% TritonX-100 in PBS solution. Cells were then incubated with primary antibody against NF-κBp65 (rabbit polyclonal, Santa Cruz, Dallas, TX, USA 1:200), IL-6 (mouse monoclonal, Abcam, 1:1000) overnight at 4 °C. After repeated rinses with PBS solution, cells were incubated with appropriate Alexa fluorogenic secondary antibodies (Invitrogen or Abcam) to detect the signal at room temperature for 1.5 h. After another set of washing, cells were mounted. The transcriptional level of IL-8 was evaluated by constructed CRT (IL-8p-d2EGFP) cells which is a destabilized enhanced green fluorescent protein (EGFP)-expressing plasmid under the control of the IL-8 promoter. All the images were captured with Zeiss inverted epifluorescence microscope (Goettingen, Germany). 

The optical density analysis to quantify fluorescence signal was performed by using CellProfiler Software (2.2.0) (Window version, http://www.cellprofiler.org). Each evaluation was conducted on five fields randomly selected for each of the target proteins.

### 4.8. Statistical Analysis

All data were expressed as mean ± SD except where indicated. Results presented are representative of at least three separate experiments using three biological replicates. *p* values were generate using Student’s t-test or one-way ANOVA, and statistical significance was considered for *p* < 0.05. Fisher protected lease significant different (FPLSD) post hoc test were used for multiple comparison.
